# Whole Genome Duplication and Enrichment of Metal Cation Transporters Revealed by *De Novo* Genome Sequencing of Extremely Halotolerant Black Yeast *Hortaea werneckii*

**DOI:** 10.1371/journal.pone.0071328

**Published:** 2013-08-15

**Authors:** Metka Lenassi, Cene Gostinčar, Shaun Jackman, Martina Turk, Ivan Sadowski, Corey Nislow, Steven Jones, Inanc Birol, Nina Gunde Cimerman, Ana Plemenitaš

**Affiliations:** 1 Institute of Biochemistry, Faculty of Medicine, University of Ljubljana, Ljubljana, Slovenia; 2 Centre of Excellence for Integrated Approaches in Chemistry and Biology of Proteins (CIPKeBiP), Ljubljana, Slovenia; 3 Department of Biology, Biotechnical Faculty, University of Ljubljana, Ljubljana, Slovenia; 4 Canada’s Michael Smith Genome Sciences Centre, BC Cancer Agency Genome Sciences Centre, Vancouver, British Columbia, Canada; 5 Department of Biochemistry and Molecular Biology, University of British Columbia, Vancouver, British Columbia, Canada; 6 Department of Pharmaceutical Sciences, University of British Columbia, Vancouver, British Columbia, Canada; University of California Riverside, United States of America

## Abstract

*Hortaea werneckii*, ascomycetous yeast from the order Capnodiales, shows an exceptional adaptability to osmotically stressful conditions. To investigate this unusual phenotype we obtained a draft genomic sequence of a *H. werneckii* strain isolated from hypersaline water of solar saltern. Two of its most striking characteristics that may be associated with a halotolerant lifestyle are the large genetic redundancy and the expansion of genes encoding metal cation transporters. Although no sexual state of *H. werneckii* has yet been described, a mating locus with characteristics of heterothallic fungi was found. The total assembly size of the genome is 51.6 Mb, larger than most phylogenetically related fungi, coding for almost twice the usual number of predicted genes (23333). The genome appears to have experienced a relatively recent whole genome duplication, and contains two highly identical gene copies of almost every protein. This is consistent with some previous studies that reported increases in genomic DNA content triggered by exposure to salt stress. In hypersaline conditions transmembrane ion transport is of utmost importance. The analysis of predicted metal cation transporters showed that most types of transporters experienced several gene duplications at various points during their evolution. Consequently they are present in much higher numbers than expected. The resulting diversity of transporters presents interesting biotechnological opportunities for improvement of halotolerance of salt-sensitive species. The involvement of plasma P-type H^+^ ATPases in adaptation to different concentrations of salt was indicated by their salt dependent transcription. This was not the case with vacuolar H^+^ ATPases, which were transcribed constitutively. The availability of this genomic sequence is expected to promote the research of *H. werneckii*. Studying its extreme halotolerance will not only contribute to our understanding of life in hypersaline environments, but should also identify targets for improving the salt- and osmotolerance of economically important plants and microorganisms.

## Introduction

Salinization of soil as a form of land degradation is rendering large areas of arable lands useless for crop cultivation and is thus an increasingly important cause of agricultural losses [1], [2]. High concentrations of salt decrease the performance of plants by disrupting all the major processes required for their growth, including photosynthesis and energy metabolism [3]. Attempts at breeding salt-tolerant lines or cultivars of crops have failed to overcome this problem and genetic engineering has yet to yield the desired results [4]. The majority of genes that have been used for this purpose to date have originated from salt-sensitive donors. Since novel sources of genes are still much needed [5], [6], more attention should be focused on organisms from natural hypersaline environments. Among these, halotolerant or halophilic fungal species are promising candidates [7]. Working with them, however, demands considerably more effort compared to conventional model organisms with sequenced genomes. Accordingly, the draft genome sequence of one such fungus, *Hortaea werneckii*, can open new avenues for experimental exploitation of its genetic resources. These may prove to be useful not only for crop improvement, but also for industrial microorganisms. For example *Saccharomyces cerevisiae* with increased tolerance to osmotic stress would be greatly beneficial in the ethanol production industry [8]. In addition to the obvious biotechnological applications, this work represents the first published genome sequence of an extremely halotolerant fungus and is thus an important step toward better understanding of eukaryotic halotolerance.

*Hortaea werneckii* is a melanised yeast-like fungus, belonging to the ascomycetous order Capnodiales. It was primarily known as a causative agent of *tinea nigra*, a superficial mycotic infection of the human skin mainly affecting the palms [9]. It was also found on salty food [10] and other low-water-activity substrates such as arid inorganic and organic surfaces [11], seawater [12] and beach soil [13]. The primary environmental ecological habitat of *H. werneckii* is probably hypersaline water in evaporite ponds of solar eutrophic salterns [14], [15] but in the salterns it was also found on wood immersed in brine [16], in biofilms on the surface of hypersaline waters, in the soil in dry evaporite ponds and in the saltern microbial mats [14]. It is one of the most salt-adaptable species known among eukaryotes as it can cope with solutions of NaCl ranging from 0% to almost saturating concentrations [15]. High adaptability and salt tolerance are properties that make *H. werneckii* a very appropriate model system for studying salt tolerance in eukaryotes.

To date, studies on *H. werneckii* have focused on various adaptations to high concentrations of NaCl and low water activity: changes in morphological features [17], membrane properties and composition [18] [19], [20], and accumulation and synthesis of compatible solutes like erythritol, arabitol, mannitol, mycosporine-glutaminol-glucoside, and glycerol as the major compatible solute [21]–[23], ion pumps and accumulation of ions [24], [25], melanisation of cell wall [22], [26], the salt-sensing signal transduction pathway(s) [27]–[30], and isoprenoid synthesis [31], [32]. These studies, together with analysing the differential gene transcription [23], [33] revealed many mechanisms that are employed by *H. werneckii* to successfully adapt to changing salinities (a proposed synthesis of all current knowledge was published by [34]). The study on the enzyme Hal2 (3′-phosphoadenosine-5′-phosphatase) provided experimental evidence that two *HAL2* homologues, identified in *H. werneckii*, could be used in improving salt tolerance of transgenic yeast strains and possibly of plants [35].

So far, however, most attempts at improving salt tolerance of plants have focused on either improving osmotic adjustment or Na^+^ exclusion, but targeting the K^+^ homeostasis was mostly neglected (reviewed in [36]). In this sense, the cation transporters of *H. werneckii* as possible transgenes for crop improvement represent an opportunity that is still completely unexplored. The cell transporter systems are of key importance in survival of high salinity environments. Systems ensuring efficient uptake and efflux of K^+^ and/or Na^+^ are highly conserved [37]. Reports describe several cases of successful expression of plant cation transporters in yeast [38]–[40] and vice versa [41], [42]. Various transporters are most extensively studied in the model organism *S. cerevisiae*. When yeast cells are exposed to high salinities, they expend energy accumulating sufficient amounts of intracellular K^+^, which is required for many physiological functions; and for maintaining low cytosolic Na^+^, which binds to and inhibits several enzymes involved in key metabolic processes in the cytoplasm [43].

At the plasma membrane the potassium uptake systems Trk1 and Trk2 [44], the potassium channel Tok1 [45], the P_i_- Na^+^ symporter Pho89 [46] and the efflux systems Ena (Na^+^-ATPases) [47]–[49] and Nha1 (Na^+^/H^+^ antiporter) [50] maintain the sufficient intracellular K^+^ amounts and the K^+^ homeostasis, exclude toxic Na^+^, preserve the membrane potential, keep the positive turgor inside the cell and cope with osmotic stress [43]. Intracellular cation/H^+^ antiporters, like vacuolar Vnx1 [51], endosomal Nhx1 [52], Golgi apparatus Kha1 [53] and mitochondrial K^+^/H^+^ exchanger system (Mdm38, Mrs7, Ydl183c) [54], [55] similarly serve to regulate the cytosolic and intraorganellar cation homeostasis and pH and modulate protein trafficking through the endosomal pathway [43]. The energy needed for the functioning of these systems comes from the plasma membrane (Pma1, Pma2) and vacuolar (Vma1) H^+^-ATPases [43]. Pma1 is the most abundant plasma membrane protein, and is responsible for establishing the electrochemical gradient of protons across the membrane that is used by secondary active symporters and antiporters [56], [57]. Vma1, on the other hand, is a multi-subunit protein complex that has an important role in energising the organellar cation/H^+^ antiporters [58]. Both are also involved in maintaining the pH homeostasis and are functionally interconnected [43]. Additional K^+^ influx systems, like K^+^- H^+^ symporter (Hak), K^+^-Na^+^ P-type ATPase (Acu), and K^+^ efflux channel Tok, were identified in the nonconventional yeasts (reviewed in [59], [60]), and were later shown to be widely present in several fungal species [61]. Importantly, fungi and plants show many similarities in the way in which the plasma membrane is energised, and K^+^ and Na^+^ are transported [36], [62], but the Na^+^-ATPases commonly present in fungi are absent in higher plants [63], [64].

The extreme conditions that define the natural habitats of *H. werneckii* demand efficient cellular mechanisms to combat all the problems that are caused by high concentrations of toxic inorganic salt ions. The remarkable ability of *H. werneckii* to not only thrive in hypersaline conditions that are lethal to a majority of other microorganisms, but also survive and grow without salt (which is not the case with, for example, halophilic Archaea), indicates the existence of unique adaptations. The aim of our study was to unravel such adaptations by making an inventory of metal cation transport systems in *H. werneckii*, and interpret them in light of its extremely adaptable and halotolerant character. Interesting gene targets for improvement of plant salt tolerance were also identified and discussed. For this purpose the genome of *H. werneckii* was sequenced, *de novo* assembled and annotated.

## Materials and Methods

### Strain and DNA/RNA Preparation

The halophilic black yeast-like fungus *H. werneckii* (strain EXF-2000) was isolated from marine solar salterns on the Adriatic coast (Slovenia) [15]. It is maintained in the Ex Culture Collection of the Department of Biology, Biotechnical Faculty, University of Ljubljana (Infrastructural Centre Mycosmo, MRIC UL, Slovenia) and in the CBS culture collection (Centraalbureau voor Schimmelcultures, the Netherlands) as strain CBS 100457. *H. werneckii* cells were grown in supplemented synthetic defined yeast nitrogen base (YNB) liquid medium (ForMedium, UK): 1.7 g YNB medium, 0.8 g complete supplement mixture (CSM), 5 g (NH_4_)_2_SO_4_, and 20 g glucose, per litre of deionised water. The medium was adjusted to pH 7.0 and to NaCl concentrations of 0%, 5%, 10%, 17% and 25% (w/v). Incubations were performed at 28°C in 500 ml Erlenmeyer flasks on a rotary shaker at 180 rpm. Inoculum cultures were grown in 25 ml YNB at the appropriate NaCl concentrations to the mid-exponential phase. Growth was monitored spectrophotometrically by optical density at 600 nm (OD_600_), cells were grown to mid-exponential phase (OD_600_ = 0.8–1.0) and harvested by centrifugation (4000×*g*; 10 min).

For DNA isolation, *H. werneckii* was grown in the YNB liquid medium without NaCl and harvested by centrifugation in the mid-exponential growth phase. The pellet was frozen in liquid nitrogen and homogenised using a mortar and pestle. The DNA was then isolated according to the protocol described by Rozman and Komel [65]. The integrity, purity, and quantity of the DNA were evaluated with Agilent 2100 Bioanalyzer (Agilent Technologies, USA) and spectrophotometrically with NanoDrop 2000 (Thermo Fisher Scientific, USA).

For RNA isolation, *H. werneckii* was grown in the YNB liquid medium with different amounts of NaCl added (0%, 5%, 10%, 17% and 25%; w/v), and harvested by centrifugation in the mid-exponential growth phase. RNA was isolated using TRI REAGENT ™ (Sigma, Germany) according to the manufacturer instructions. Possible DNA contaminations were degraded with deoxyribonuclease I (Thermo Fisher Scientific - Fermentas, Lithuania) and the RNA was additionally cleaned with Qiagen RNeasy MinElute Clean up Kit (Qiagen, USA). The integrity and purity of the RNA was evaluated with Agilent 2100 Bioanalyzer (Agilent Technologies, USA).

### Genome Sequencing and Assembly

A single sequencing library with an input fragment size of 400-bp was constructed using the NEBNext DNA sample prep kit (New England Biolabs Ltd). Paired-end 75-bp reads were generated on an Illumina GAIIx DNA sequencer (Illumina Inc). One lane of Illumina GAIIx 75-bp paired-end reads, yielding 5.7 Gbp of sequence and an estimated 110-fold coverage of the genome, was assembled using ABySS 1.2.1. To assemble reads in low-coverage regions, the reads were first assembled setting the de Bruijn graph parameter*k* to a small value, *k = 25*. The reads were then reassembled at a larger value of *k*, *k = 60*, including the *k = 25* contigs as additional sequence. The paired-end assembly parameters of ABySS were set to *s = 150* and *n = 10*. All other parameters were set to their default values. This Whole Genome Shotgun project has been deposited at DDBJ/EMBL/GenBank under the accession AIJO00000000. The version described in this paper is the first version, AIJO01000000. The raw short reads are accessible in the NCBI Sequence Read Archive under the accession number SRR866616.

### Gene Prediction and Annotation

Automatic prediction of the genes was made by first determining the putative open reading frames (ORFs) with the MAKER genome annotation pipeline version 2.25 [6665] on a computer running Biolinux 6 [67], [66]. Several sets of data were used as evidence for annotation: all *H. werneckii* transcripts available in GenBank, transcripts of *Aureobasidium pullulans* (our unpublished data), *Pyrenophora tritici-repentis* [68] and *Mycosphaerella graminicola* [69] as well as all proteins from the UniProtKB/Swiss-Prot database. *Pezizomycotina* were used as a model for repeat masking. Three gene predictors were used: Augustus (trained for *Neurospora crassa*), GeneMark (self-trained with GeneMark.hmm-ES) and Snap (trained with 12 sequential outputs of the Maker pipeline). Functional annotation of the ORFs was performed with the Blast2GO software [70]. Basic analyses of the predicted genes and proteins were performed with the EMBOSS suite [71]. Pfam domains of predicted proteins were identified with a stand-alone Pfam Scanner and a database downloaded on 30. 1. 2013 [72]. For comparison purposes the same was done for *Saccharomyces cerevisiae* and *M. graminicola*. The results were used to determine the number of proteins with a given domain in each of the proteomes (Table S1).

### Gene Duplication Analyses

Gene duplications were detected by two different methods. Predicted proteins were aligned to the genome with Exonerate version 2.2.0 using protein2genome model [73] and limiting the reported hits to those above the certain percent of maximal score obtainable for that query. Additionally, an all-against-all protein sequence similarity search of *H. werneckii* proteins to a *H. werneckii* protein database was performed by blastp included in the BLAST 2.2.25+ [74]. The number of hits was counted for each query. For comparison purposes the same analysis was also performed for proteomes/genomes of *Mycosphaerella graminicola* [69] and *Saccharomyces cerevisiae* (SGD project. http://www.yeastgenome.org/download-data/(22. 5. 2012)). The numbers of shared and unique proteins between *H. werneckii*, *M. graminicola* and *S. cerevisiae* were determined by all-against-all blast of their whole proteomes for all possible pairs of species.

### Manually Curated Gene Annotations

To identify all alkali metal cation transporters coded in the *H. werneckii* genome, the databases of automatically annotated *H. werneckii* ORFs were searched for homologues of the already identified *S. cerevisiae* transporters or transporters identified in unconventional yeasts [43], using blastn and blastx algorithms, respectively. From the list of results, only hits with e-values lower than 1e^−6^ were analysed further. The analysis retrieved not only proteins from the same transporter group as the query, but also a significant amount of transporters from other groups; however, to avoid missing possible highly divergent transporter homologues the cut-off e-value was not lowered. Proteins that were retrieved more than once were kept only in one copy for further analyses. ORFs were then re-aligned to the genomic sequence and, where necessary, the translation start and stop sites were manually corrected according to the comparison with the N-terminal and C-terminal ends of homologues from other fungi. The position of introns was confirmed by manually identifying conserved intron donor and acceptor sites. Where we encountered two or more contiguous ORFs that could be determined as parts of the same gene with large certainty, these fragments were merged into a single entry. The same protocol was also used for the analysis of the *H. werneckii* MAT locus, where *S. cerevisiae* and *M. graminicola* Mat1-1-1 and Mat1-2-1 proteins were used as sequence queries.

### Gene Phylogeny Reconstruction

Amino acid sequences of the manually curated *H. werneckii* homologues of the *S. cerevisiae* alkali metal cation transporters together with the *S. cerevisiae*, *M. graminicola* and *Crypotococcus neoformans* homologous transporters, were used to build phylogenetic trees. The same analysis was performed with proteins that were identified by blastp (e-value cut-off 10^−6^) in the predicted proteome of *H. werneckii* using the homologues of all known P-type ATPases from *S. cerevisiae* as queries. Homologues from *Ajellomyces dermatitidis, Leptosphaeria maculans*, *Mycosphaerella graminicola*, and *Paracoccidioides brasiliensis* were also included in the phylogenetic analysis. Protein sequences were aligned using the L-INS-i method in the MAFFT software [75]. ProtTest 3.2.1 [76] was used to estimate the most appropriate model of protein evolution. The gene trees were generated with the PhyML 3.0 software [77] with aLRT implementation, for the calculation of branch supports as Chi2 based support. The analyses were run using the VT model of evolution for a Nhx proteins and all P-type membrane transporters and LG model for all the rest. ProtTest estimate of alpha parameter of gamma distribution of six substitution rate categories was used, and in case of Ena and Nha proteins, also the determined proportion of invariable sites. For comparison, a second set of trees (not shown) was generated by applying a maximum parsimony method as implemented in the Mega software version 5.05 [78].

### Gene Transcription Analyses

First strand cDNA was synthesized from 1 µg of total *H. werneckii* RNAs using RevertAid™ H Minus First Strand cDNA Synthesis Kit and random hexamer primer (Thermo Fisher Scientific - Fermentas, Lithuania) according to the manufacturer instructions. cDNA concentration was measured spectrophotometrically with NanoDrop 2000 (Thermo Fisher Scientific, USA). Approximately 100 ng of cDNA (10 ng for reference gene 28S rRNA) was used as a template for quantitative reverse transcription PCR with oligonucleotides specific for the genes under investigation. The primer sequences are given in the Table S2. The thermal profile of the reaction was as follows: 10 min at 95°C, 45 cycles consisting of 15 s at 95°C, 30 s at 50–60°C and 15 s at 72°C, followed by a dissociation curve (15 s at 95°C, then 60 s at 60°C and 15 s at 95°C, ramping at 0.3°C/sec). The reaction mix was prepared using the Power SYBR Green PCR Master Mix (Life Technologies, USA), according to the manufacturer instructions, in 10 µl of total reaction volume, primer concentration was 300 nM. The reactions were performed in a StepOnePlus Real-Time PCR System (Life Technologies, USA), and analyzed with StepOne v2.2.2 software (Life Technologies, USA) using the standard curve method. Relative standard curves (PCR reaction efficiency) were determined by amplifying five 10-fold serial dilutions of control cDNA. Quantification cycle (C_q_) values for our genes of interest were normalised to the quantification cycle of 28S rRNA fragment (reference gene), the transcription of which remains unchanged under different environment conditions [18]. The difference in C_q_ values representing relative mRNA level values between the target gene and the reference gene was calculated, and these values of the different samples were compared directly.

## Results and Discussion

### Sequencing and Assembly of the *Hortaea werneckii* Genome

**Figure 1:**
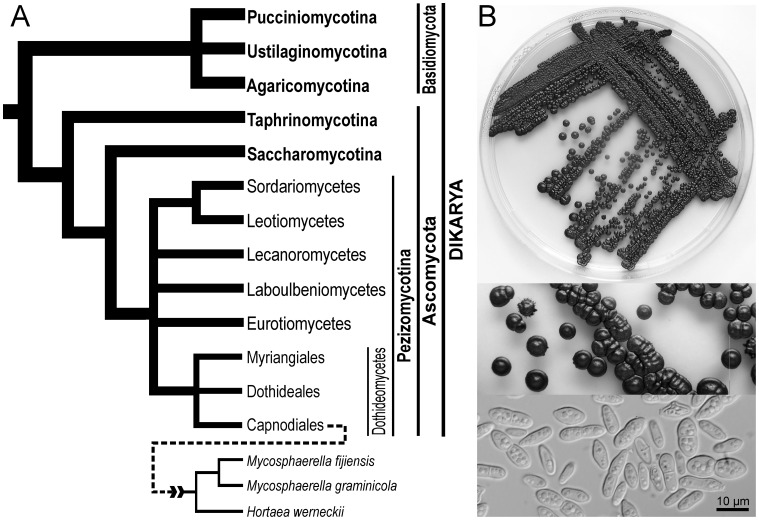
Hortaea werneckii. **A.** Schematic phylogenetic tree showing the evolution of major fungal groups [128] and the position of *H. werneckii*, together with two other *Mycosphaerella* spp. with sequenced genomes. **B.** *H. werneckii* colonies on agar after two weeks at room temperature (above and middle) and microscopic image of the cell suspension at 1000× magnification (below).

In the present study, we have sequenced the genome of the extremely halotolerant black yeast *Hortaea werneckii* using an Illumina GAIIx sequencer. A similar sequencing strategy was reported to be sufficient to produce an assembly covering most of the protein-coding genome for the fungus *Sordaria macrospora* (genome size 40 Mb) [79]. The total assembly size of the *H. werneckii* genome is 51.6 Mb (Table 1). The average genome coverage was 110× and the assembly process yielded 12620 contigs. The assembled sequence was deposited in the public genome database DDBJ/EMBL/GenBank under the accession number AIJO01000000. The genomic G+C content is relatively high at 54% and is even higher in the coding regions (56%). Of eighteen plant-associated fungi also belonging to *Dothideomycetes* (Figure 1), which were compared by Ohm et al. [68], only *Cladosporium fulvum* and *Mycosphaerella fijiensis* genomes exceed this size (61.11 Mb and 74.14 Mb, respectively), largely due to a substantial amount of repetitive sequences (44.44% and 39.50% of the genomes, respectively). Even in the seven species belonging to the same order as *H. werneckii* (Capnodiales) the genome sizes are very variable (from 21.88 to 74.12 Mb). In *H. werneckii*, however, the proportion of repetitive sequences remains low at only 1.02%, despite its large genome size. The average size of sixteen other genomes studied by Ohm et al. [68] is considerably smaller (35.33 Mb).

**Table 1:** Assembly and gene statistics for the *Hortaea werneckii* genome.

**Coverage**	∼ 110x
**Assembly size** (Mb)	51.6
**Contigs** (bp)	12 620
Contig N_50_ (bp)	8 187
Contig max (bp)	71 563
**Scaffold** (bp)	11 193
Scaffold N_50_ (bp)	9 268
Scaffold max (bp)	71 563
**Predicted protein-coding genes**	23 333
Predicted proteins with at least one match in InterProScan	22 164
Predicted proteins with blast hits in nr GenBank database (e-value <10^−6^)	20 071
Predicted proteins annotated with Blast2GO	16 103
**GC content** (%)	54
GC content in the coding sequence (%)	56
**Repeat content** (%)	1.02
Retroelements (%)	0.41
DNA transposons (%)	0.09
Simple repeats (%)	0.22
Low complexity (%)	0.26

**Figure 2:**
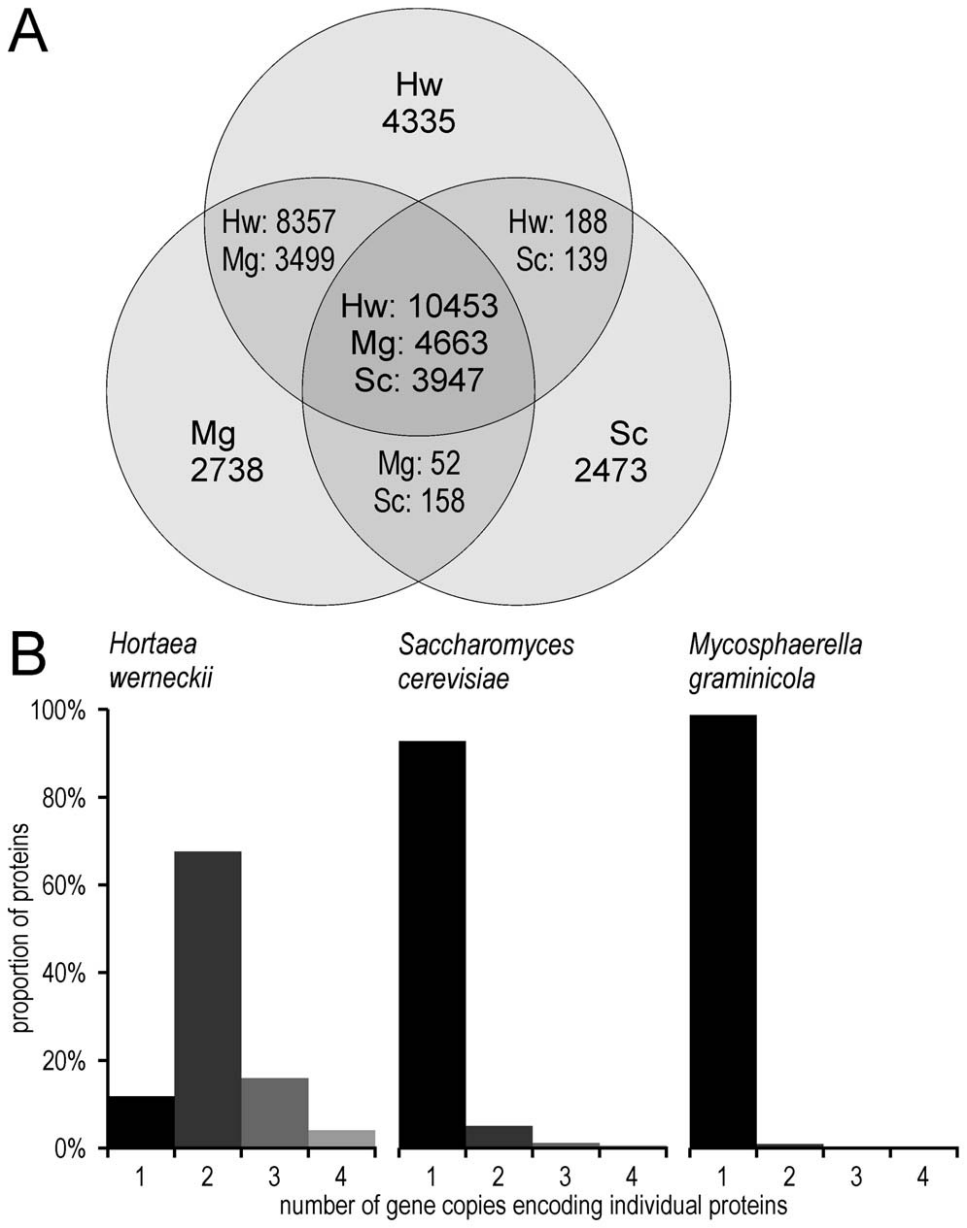
Proteomes of *Hortaea werneckii*, *Mycosphaerella graminicola* and *Saccharomyces cerevisiae*. **A.** Shared and unique proteins of three fungal species as determined by all-against-all blastp. Abbreviations used are. Hw: *H. werneckii*, Mg: *M. graminicola* and Sc: *S. cerevisiae*. **B.** Number of duplicated proteins. All predicted proteins were aligned to the genome by Exonerate and the number of possible alignment locations was counted for each protein. The proportion of proteins encoded by a certain number of gene copies are represented as columns of different heights (separately for each genome).

Despite the great variability of genome sizes in the 18 above mentioned fungi, the differences in the number of predicted genes were much smaller. On average each species contained 11955 genes (minimum 9739, maximum 14127) [68], but in *H. werneckii* this was almost twice as large (23333). Of these relatively few (18.6%) had no blast hits in the proteomes of *Saccharomyces cerevisiae* or *Mycosphaerella graminicola* (one of the phylogenetically closest species with sequenced genomes [69]) or both (Figure 2A) and 14.0% had no hits in the GenBank non-redundant database (e-value cut-off 10^−6^). This abundance of genes could be explained by large scale genomic duplications, but the relatively poor assembly of the *H. werneckii* genome could also significantly contribute to the overestimation. The large number of contigs could result in fragmented genes, later falsely identified as individual proteins. However, our manual analysis of a subset of transporter genes strongly suggested that the large number of predicted genes was not primarily the consequence of the assembly quality, as only a small subset of genes were fragmented. Instead, the large number of genes appears to be the result of a relatively recent whole genome duplication (WGD), yielding two nearly identical copies of almost every protein-encoding gene in *H. werneckii*. The large number of obtained contigs was therefore a consequence rather than the cause of the apparent duplications (due to difficulties in assembling the reads across a large number of highly similar stretches of DNA).

### Evidence for Whole-gene Duplication

To investigate if the large number of predicted proteins in *H. werneckii* is due to large-scale gene duplication, we analyzed its proteome with all-against-all blastp and aligning the proteins back to the genome (Figure 2B). Using the same parameters of analyses that detected less than 10% of duplicates in the genomes of *S. cerevisiae* and *M. graminicola*, we determined that nearly 90% of *H. werneckii* genes exist in at least two copies. Even for the relatively small fraction of single-copy proteins, we found a disproportionately large number of falsely predicted proteins: only 46.8% of them had blast hits in the non-redundant GenBank database (e-value cut-off 10^−6^), compared to 91.2% of other genes. This discovery was not entirely surprising, since previous studies of several individual genes from *H. werneckii* already noted that a majority were present in two copies [80]. In most cases, the transcription of both gene copies was salt dependent, but the transcription profiles differed [21], [29]. As a consequence of this WGD *H. werneckii* can benefit from the potential advantages of a large genetic redundancy even though it is formally haploid, as determined by the present study. At this point it is difficult to assess the adaptive value of this phenomenon, and whether it contributed to the ability of the fungus to adapt to such a wide range of environmental salinities.

Studies on other fungi indicate that this may well be the case. WGDs have been observed in several other fungal species and have been extensively studied in *S. cerevisiae* [81]. An ancient polyploidisation event occurred in the phylogenetic lineage leading to this yeast [82], yet in its contemporary natural population a large portion of triploid and tetraploid strains co-exists with diploids [83]. Experimental evolution studies have reported a very fluid nature of ploidy levels in this and other species [84], [85]. Strains with less common genome sizes tend to return to their usual ploidy over time even if this appears to be counterproductive. In *S. cerevisiae* for example, haploid strains theoretically suffer from the lowest mutation load and have also been shown to adapt faster to stressful concentrations of salt and several other conditions [86], [87]. Nevertheless it is its diploid state that appears to have fine-tuned for optimal fitness over evolutionary time. Altered gene expression patterns and altered cell geometries that are associated with other ploidy levels [88] may simply be suboptimal and therefore selected against. In addition, in the short term, diploid individuals are better able to mask deleterious mutations, which is another possible reason why they prevail over haploids in both normal and even faster in stressful conditions [86]. Even in the case of tetraploid *S. cerevisiae* strains, which normally tend to decrease in genome size toward a diploid state, this process is slower in a salt-stressed compared to a normal medium [84]. Another experimental evolution study suggested that adaptation of *S. cerevisiae* to stressful concentrations of salt is, among other changes, associated with increases in genome size [89]. Ploidity increase in *S. cerevisiae* has also been described to act as protection to ultraviolet radiation [90]. We cannot rule out this possibility even in the case of *H. werneckii*, since in shallow ponds of hypersaline water, its presumed natural habitat, it is exposed to substantial amounts of solar radiation.

### *MAT* Loci of the Heterothallic *Hortaea werneckii*

**Figure 3:**
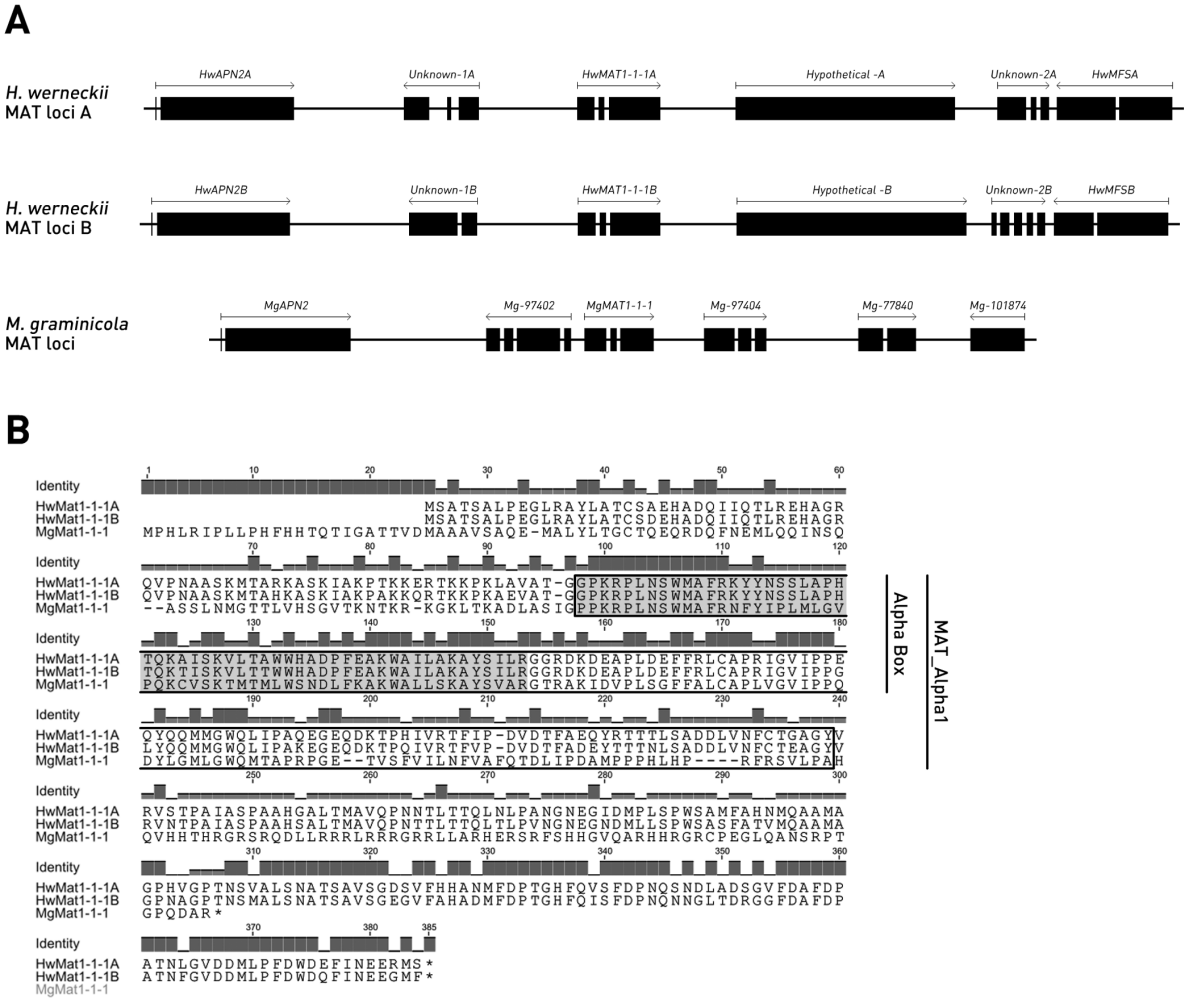
Heterothallic *Hortaea werneckii MAT* loci. **A.** Configuration of the *MAT* loci and adjacent genes in *H. werneckii* compared to *Mycosphaerella graminicola*. Arrows indicate the length and direction of transcription of the gene. Gene names are indicated above the gene model (black for exones, white for intrones). The models are drawn in scale (1 cm per 1000 nucleotides). **B.** Amino acid sequence alignment of HwMat1-1-1A (KC961394), HwMat1-1-1B (KC961395) and MgMat1-1-1 (XP_003847598.1) proteins. The MAT_Alpha1 conserved domain (PF04769) is indicated by a box, whereas the Alpha Box (PS51325, Prosite) is designated by dark grey.

*H. werneckii* has a complex asexual cycle, where changes in nutrition, cell number or temperature mediate conversion between the yeast and hyphae cell morphology [91]. Still, no sexual cycle has been described so far. The *H. werneckii* draft genome sequence therefore offered the opportunity to gain insight into the genetic information on the mating type/types and on the mating strategy. Previous investigations of the ascomycete sexual reproduction have established that this process is determined by the presence of different arrangements of mating-type (*MAT*) genes at one or more MAT loci [92]. Two mating types exist, the idiomorph MAT1-1, which contains the *MAT1-1-1* gene encoding a protein with an alpha1 domain; and MAT1-2 idiomorph, which contains the *MAT1-2-1* gene encoding the high mobility group (HMG) domain protein [92]. The *H. werneckii* related genus *Mycosphaerella* contains numerous self-incompatible (heterothallic) species, coding only for one of the idiomorphs; and self-compatible (homothallic) species, coding for both mating types [93]. We used *M. graminicola* alpha1 domain (Mat1-1-1, XP_003847598.1) and HMG domain (Mat1-2-1, ABH04241.1) containing proteins to screen the *H. werneckii* genome for existing mating type loci with tblastn. We identified two genes, both encoding a homologue of the *M. graminicola MAT1-1-1* gene (Figure 3), and designated them *HwMAT1-1-1A* (KC961394) and *HwMAT1-1-1B* (KC961395). *HwMAT1-1-1A* gene is 1196 bp long and contains two introns, the 58 bp long intron and the 69 bp long intron, which lies inside the alpha1 domain (Figure 3A). The *HwMAT1-1-1B* gene is 88.7% identical to *HwMAT1-1-1A* in the nucleotide sequence, and also contains two introns, 58 bp and 56 bp long, the last one again located inside the alpha domain (Figure 3A). Both *HwMAT1-1-1* homologues translate into 358 aa long proteins (HwMat1-1-1A and HwMat1-1-1B) with 141 aa long alpha1 domain (PF04769), and have an overall amino acid (aa) sequence identity of 87.5% (Figure 3B). When compared to MgMat1-1-1, the overall aa sequence identity of HwMat1-1-1 homologues was only around 29%, but the identity increased to around 43% if comparing only the alpha1 domains. Importantly, no homologues of the *M. graminicola* Mat1-2-1 protein were found in *H. werneckii*, indicating that this species is heterothallic, and needs to mate with the strain coding for the opposite Mat1-2-1 homologue for sexual reproduction.

As *H. werneckii* MAT1-2 idiomorph has not yet been identified, we cannot determine the borders and the length of the MAT1-1 idiomorph. Based on comparison to the related *M. graminicola* MAT1-1 idiomorph [93], we can anticipate that HwMAT1-1 idiomorphs contain only one gene, the *HwMAT1-1-1A* or *HwMAT1-1-1B* (Figure 3A). This one gene – one idiomorph structure has also been found in other heterothallic Loculoascomycetes [92]. When analysing the 5′ and 3′ flanking regions of the *H. werneckii* MAT1-1 idiomorphs, we identified two genes upstream (*HwAPN2*, *Unknown-1*) and three genes downstream (*Hypothetical protein*, *Unknown-2*, *HwMFS*) of the idiomorph (Figure 3A). Genes *HwAPN2A* (KC961396) and *HwAPN2B* (KC961397) code for DNA lyase and contain the Ape2-like_AP-endo domain (cd09088), typical for the Ape2-like subfamily of the ExoIII family purinic/apyrimidinic endonucleases. AP endonucleases participate in the DNA base excision repair pathway, and have 3′–5′ exonuclease, 3′-deoxyribose phosphodiesterase, 3′-phosphatase and occasionaly nonspecific DNase activities [94]. HwApn2A and HwApn2B are around 60% identical to the MgApn2 and the *APN2* genes assume the same position relative to the *MAT1-1-1* homologue in *H. werneckii* and *M. graminicola* chromosome fragment (Figure 3A). The hypothetical proteins (Figure 3A) contain the conserved putative integral membrane protein region (PF10296), indicating that these are membrane proteins. Genes *HwMFSA* (KC961398) and *HwMFSB* (KC961399) code for the Major Facilitator Superfamily (MFS) transporters, responsible for transport of small solutes in response to chemiosmotic ion gradients. HwMfsA and HwMfsB are 95% identical in aa sequence. No *MFS* homologue is found in *M. graminicola* idiomorph containing genom fragment, at a similar position relative to *MgMAT1-1-1* (Figure 3A).

Interestingly, both HwMAT1-1 and MgMAT1-1 containing genome fragments show strong conservation with respect to the relative positions of genes and orientation of transcription, whereas the gene sequence is not conserved (Figure 3A). The only two exceptions are the *APN2* and the *MAT1-1-1* gene homologues, where also the sequence is conserved to some extent. A similar arrangement was also shown for the heterothallic *Neurospora* species [95].

### Alkali Metal Cation Transporters in *Hortaea werneckii*

*H. werneckii* natural habitats are thalassohaline hypersaline environments, which originated by the evaporation of seawater [34]. They are characterized by high and dynamically changing concentrations of dominating sodium and chloride ions, and the pH is near neutral to slightly alkaline. These extreme environments are frequently accompanied by other types of stress, such as high temperatures, high UV radiation, low oxygen concentrations, or generally low nutrient availability with occasional peaks in abundance [34], [96], [97]. A typical example of such environments is the solar saltern, where seawater is evaporated to produce sea salt and the hypersaline brine. Brines derived from seawater contain relatively high concentrations of Ca^2+^ and can remain approximately neutral even after extensive evaporation because the molarity of Ca^2+^ always exceeds that of CO_3_^2−^, which is part of the carbonate buffer system of the aquatic environment [98].

For organisms living in such environments, maintaining intracellular cation homeostasis, i.e., maintaining rather high and stable K^+^ content and eliminating toxic Na^+^ ions [36], [43], is crucial for survival. *H. werneckii* maintains very low amounts of internal Na^+^, even when grown in environments with high NaCl concentrations; although the K^+^/Na^+^ ratio does fall with increasing salinities [25]. Altogether 2208 (9.6%) of *H. werneckii* predicted proteins were assigned the GO term “transport” (biological processes) (Figure S1), similarly to what was shown for Hemiascomecete yeasts (10%) [37]. To get an insight into the transport systems responsible for maintaining cation homeostasis in extremely halotolerant *H. werneckii*, we searched the databases of predicted *H. werneckii* ORFs and proteins for homologues of metal cation transporters from *S. cerevisiae* and nonconventional yeasts [43], [59]. The predicted *H. werneckii* transporters were assigned standard names of the closest orthologues from *S. cerevisiae* according to the Saccharomyces Genome Database (SGD) [99]. The prefix “Hw” for *Hortaea werneckii* was added, the genes were consecutively numbered, and in case of paralogues resulting from a recent (WGD) duplication, this was indicated by a suffix of letters “A” or “B”. Similarly, we searched the proteomes of the *M. graminicola* [100] and the *Cryptococcus neoformans* var. neoformans JEC21 [101], using the same list of transporter proteins for query sequences as for *H. werneckii*.

We identified homologues of all characterized *S. cerevisiae* plasma membrane (Trk1, Trk2, Tok1, Nha1, Ena proteins, Pho89) and intracellular cation transporters (Kha1, Mrs7, Vnx1, Nhx1) (Table 2) as well as homologues of the H^+^ -ATPases (Pma paralogues, V-ATPase complex) important for supplying the energy to the secondary transporters (Table 3). Interestingly, no homologues of the Hak1 and Acu1 transporters, found in nonconventional yeasts [59], [60] and in many other Ascomycete fungi [61], were identified in the predicted *H. werneckii* proteome. On the other hand, these two transporters are present in the closely related *M. graminicola*, in addition to all above mentioned *S. cerevisiae* transporters.

**Table 2:** Alkali metal cation transporter homologues in extremly halotolerant *Hortaea werneckii*.

Cation transporters		*H. werneckii*	Accesion No.	Lenght (aa)
K^+^ channel	Plasma mem.: K^+^ uptake	HwTrk1A	KC961318	787^a^
		HwTrk1B	KC961319	788^a^
		HwTrk2A	KC961320	586^a^
		HwTrk2B	KC961321	690^a^
		HwTrk3A	KC961322	663
		HwTrk3B	KC961323	663
		HwTrk4A	KC961324	702^a^
		HwTrk4B	KC961325	771
	Plasma mem.: K^+^ efflux	HwTok1A	KC961326	684
		HwTok1B	KC961327	684
		HwTok2A	KC961328	769
		HwTok2B	KC961329	769
K^+^, H^+^ antiporter	Golgi: K^+^ uptake	HwKha1A	KC961330	882
		HwKha1B	KC961331	897
	Mitochondria: K^+^ uptake	HwMrs1A	KC961332	560
		HwMrs1B	KC961333	560
Na^+^/K^+^, H^+^ antiporter	Plasma mem.: Na^+^/K^+^ efflux	HwNha1A	KC961334	1029^a^
		HwNha1B	KC961335	860^a^
		HwNha2A	KC961336	496
		HwNha2B	KC961337	496
		HwNha3A	KC961338	672
		HwNha3B	KC961339	672
		HwNha4A	KC961340	604
		HwNha4B	KC961341	604
	Vacuole: Na^+^/K^+^ uptake	HwVnx1A	KC961342	1223
		HwVnx1B	KC961343	942^a^
		HwVnx2A	KC961344	794
		HwVnx2B	KC961345	718^a^
		HwVnx3A	KC961346	307^b^
		HwVnx3B	KC961347	307^b^
		HwVnx4A	KC961348	521
		HwVnx4B	KC961349	521
	Endosome: Na^+^/K^+^ uptake	HwNhx1A	KC961350	702
		HwNhx1B	KC961351	698
Na^+^/K^+^ - ATPase	Plasma mem.: Na^+^/K^+^ efflux	HwEna1A	KC961352	1071
		HwEna1B	KC961353	1070
		HwEna2A	KC961354	936^a^
		HwEna2B	KC961355	936^a^
Na^+^, P_i_ simporter	Plasma mem.: Na^+^ uptake	HwPho1A	KC961356	579
		HwPho1B	KC961357	579
		HwPho2A	KC961358	246^b^
		HwPho2B	KC961359	302^b^
		HwPho3A	KC961360	367^b^
		HwPho3B	KC961361	391^b^

Cation transporter homologues in *H. werneckii* were identified by blast searches of *H. werneckii* ORF and protein databases with the following *S. cerevisiae* S288C protein queries: Trk1 (YJL129C), Trk2 (YKR050W), Tok1 (YJL093C), Kha1 (YJL094C), Mrs7 (YPR125W), Nha1 (YLR138W), Vnx1 (YNL321W), Nhx1 (YDR456W), Ena1 (YDR040C), Ena2 (YDR039C), Ena5 (YDR038C) and Pho89 (YBR296C).

a A part of N-terminal or C-terminal protein sequence might be missing.

b The C-terminal part of the protein sequence is not included in the estimation of lenght, as the overlap of the 5′ and 3′ ORFs coding the gene was to short to merge them unumbigously.

**Table 3:** Homologues of H^+^ -ATPases in extremly halotolerant *Hortaea werneckii*.

H^+^ -ATPases	Subunit	*H. werneckii*	Accesion No.	Lenght (aa)
Plasma mem.: H^+^ efflux		HwPma1A	KC961362	977
		HwPma1B	KC961363	977
		HwPma2A	KC961364	1005
		HwPma2B	KC961365	1005
Vacuole: H^+^ uptake	V_1_ subunit A	HwVma1A	KC961366	579
		HwVma1B	KC961367	579
	V_1_ subunit B	HwVma2A	KC961368	287^a^
		HwVma2B	KC961369	518
	V_1_ subunit C	HwVma5A	KC961370	394
		HwVma5B	KC961371	394
	V_1_ subunit H	HwVma13A	KC961372	136^b^
		HwVma13B	KC961373	402^a^
	V_1_ subunit E	HwVma4A	KC961374	231
		HwVma4B	KC961375	231
	V_1_ subunit G	HwVma10A	KC961376	117
		HwVma10B	KC961377	118
	V_1_ subunit D	HwVma8A	KC961378	270
		HwVma8B	KC961379	270
	V_1_ subunit F	HwVma7A	KC961380	127
		HwVma7B	KC961381	124
	V_0_ subunit a	HwVph1A	KC961382	865
		HwVph1B	KC961383	511^b^
	V_0_ subunit d	HwVma6A	KC961384	865
		HwVma6B	KC961385	511^b^
	V_0_ subunit c	HwVma3A	KC961386	160
		HwVma3B	KC961387	41^b^
	V_0_ subunit c’	HwVma11A	KC961388	162
		HwVma11B	KC961389	162
	V_0_ subunit c”	HwVma16A	KC961390	202
		HwVma16B	KC961391	202
	V_0_ subunit e	HwVma9A	KC961392	83
		HwVma9B	KC961393	83

Homologues of H^+^ -ATPases in *H. werneckii* were identified by blast searches of *H. werneckii* ORF and protein databases with the following *S. cerevisiae* S288C protein queries: Pma1 (YGL008C), Pma2 (YPL036W), Vma1 (YDL185W), Vma2 (YBR127C), Vma5 (YKL080W), Vma13 (YPR036W), Vma4 (YOR332W), Vma10 (YHR039C-A), Vma8 (YEL051W), Vma7 (YGR020C), Vph1 (YOR270C), Stv1 (YMR054W), Vma6 (YMR054W), Vma3 (YEL027W), Vma11 (YPL234C), Vma16 (YHR026W) and Vma9 (YCL005W-A).

a A part of N-terminal or C-terminal protein sequence is possibly missing.

b The C-terminal part of the protein sequence is not included in the estimation of the lenght, as there was no overlap between the 5′ and 3′ ORFs coding the gene.

#### Plasma membrane cation transporters

Fungi in general, including *H. werneckii*, live in environments with highly variable potassium concentrations. Nevertheless, K^+^ is present in all cells at relatively high concentrations, compared to other cations, and it is crucial for several basic physiological functions, like osmotic regulation, protein synthesis and enzyme activation [102]. Another consequence of regulated K^+^ uptake and efflux across the membrane is also the maintenance of the plasma membrane potential [103]. High sodium concentrations in the environment disturb K^+^ homeostasis, as Na^+^ intrudes into the cell and causes lowering of K^+^ intracellular concentration, because cells have to maintain electroneutrality. While extracellular sodium causes severe osmotic stress, high intracellular concentrations of Na^+^ interfere with growth by inhibiting many important enzymatic functions [43]. Keeping a low intracellular sodium concentration and a high intracellular K^+^/Na^+^ ratio is therefore crucial for functioning of the organism [36]. In *S. cerevisiae*, transporters maintaining high intracellular K^+^ concentrations are high affinity K^+^ channels Trk1 and Trk2, involved in potassium uptake [44], and three different transporters involved in potassium efflux: membrane depolarization activated K^+^ channel Tok1 [45], Ena1-5 ATPases [47]–[49] and Na^+^/K^+^ antiporter Nha1, which were first identified as Na^+^ efflux systems [50]. Ena P-type ATPases couple ATP hydrolysis to export Na^+^ (or K^+^) from the cells at alkaline pH, whereas antiporter Nha1 uses an H^+^ gradient to energise the efflux at acidic external pH values. There is no single specific uptake transporter for sodium in *S. cerevisiae*, so it is proposed that besides K^+^, Trk1 also transports Na^+^, although with a much lower affinity. Additionally, at alkaline pH, symporter Pho89 catalyzes a sodium-dependent phosphate uptake (reviewed in [43]).

**Figure 4:**
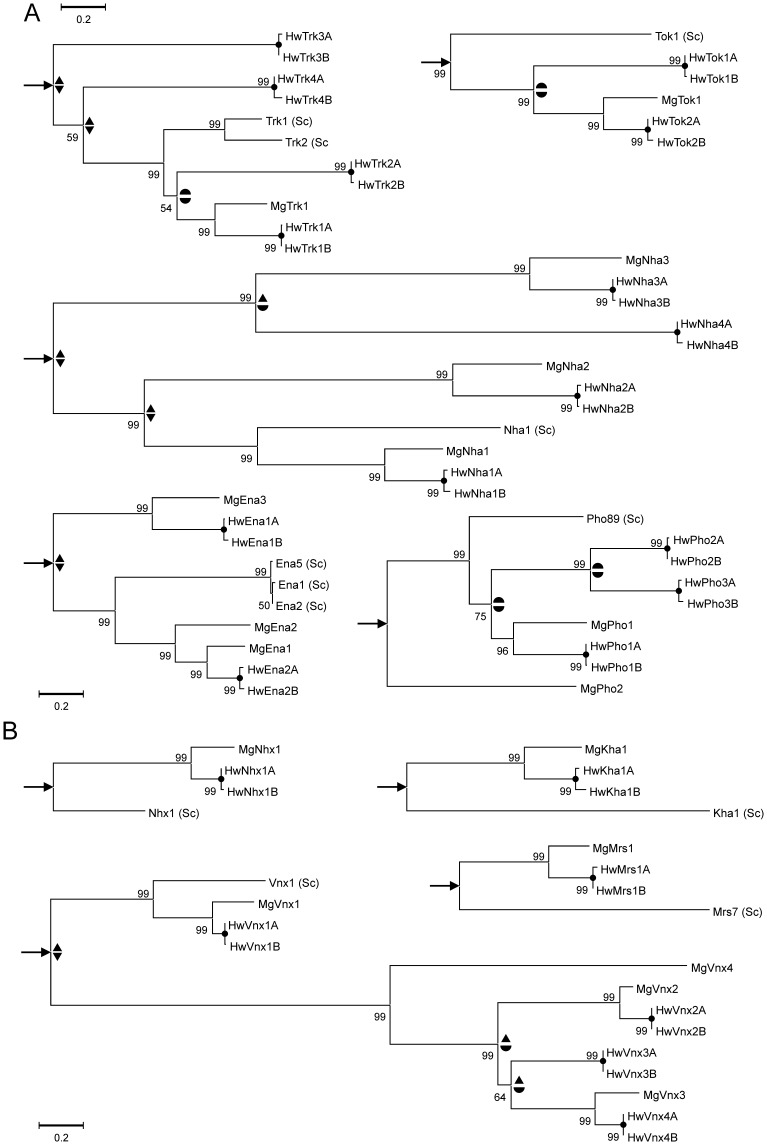
Gene trees of various membrane transporters of inorganic ions from *Hortaea werneckii* (Hw), *Mycosphaerella graminicola* (Mg) and *Saccharomyces cerevisiae* (Sc). The trees were rooted with homologous proteins from *Cryptococcus neoformans* and the root location is marked with an arrow. Putative gene duplications leading to the present diversity of these genes in *H. werneckii* are marked with different symbols: two triangles (duplications that happened before the separation of *S. cerevisiae* and *H. werneckii* ancestors), two half-circles (duplications after the separation of *S. cerevisiae* and *H. werneckii* ancestors, but before the separation of *H. werneckii* and *M. graminicola*), a combination of triangle and half circle (duplications before the separation of *H. werneckii* and *M. graminicola* but unclear regarding to separation from *S. cerevisiae* ancestor) and with circles on the bifurcation (recent duplications presumably resulting from a whole genome duplication). **A.** Plasma membrane transporters. **B.** Transporters located on internal membranes. The following *S. cerevisiae* transporters (Trk1 (YJL129C), Trk2 (YKR050W), Tok1 (YJL093C), Nha1 (YLR138W), Ena1 (YDR040C), Ena2 (YDR039C), Ena5 (YDR038C), Pho89 (YBR296C), Nhx1 (YDR456W), Kha1 (YJL094C), Vnx1 (YNL321W), Mrs7 (YPR125W)) and its *H. werneckii* (Table 2) and *M. graminicola* (Trk1: XP_003850012.1; Tok1: XP_003847595.1; Nha1: XP_003855439.1, XP_003856011.1 and XP_003855492.1; Ena1: XP_003852150.1, XP_003854801 and XP_003850456.1; Pho89: XP_003852378.1 and XP_003849212.1; Nhx1: XP_003850315.1; Kha1: XP_003852156.1; Vnx1:, XP_003854352.1, XP_003853630.1, XP_003849439.1 and XP_003852229.1; Mrs7: XP_003852324.1) homologues were included in the phylogenetic trees. Homologoues from *C. neoformans* (Trk1: XP_570017.1 and XP_569339.1; Tok1: XP_568987.1 and XP_568988.1; Nha1: XP_569560.1; Ena1: XP_572412.1, XP_568029.1 and XP_570160.1; Pho89: XP_568082.1; Nhx1: XP_570596.1; Kha1: XP_571501.1; Vnx1: XP_569752.1; Mrs7: XP_569566.1) were used as outgroups. The location of the root on the trees is marked by arrows.

In *H. werneckii* we identified 8 homologues of the Trk1 and Trk2 K^+^ channels (Table 2), each containing the conserved TrkH domain (PF02386) typical for cation transport proteins. In general, they show low similarity to Trk1 protein, but the amino acid (aa) sequence identity increases in the TrkH domain, the value falling between 37.4% and 49.6%, depending on the *H. werneckii* homologue. The expansion of the Trk channels in *H. werneckii* was also confirmed by the comparative analyses of the PFAM domains (Table S1), as PF02386 domain in *H. werneckii* was enriched 8× relative to *M. graminicola* and 4× relative to *S. cerevisiae*. Phylogeny of Trk proteins from *H. werneckii*, *M. graminicola* and *S. cerevisiae*, rooted with *C. neoformans* homologue (Figure 4A) indicates that two duplications of *H. werneckii* Trk channels happened before the separation of the *S. cerevisiae* and *H. werneckii* ancestors, but one gene copy was later lost in *S. cerevisiae* and *M. graminicola*. An additional duplication occurred later, but before the separation of the *H. werneckii* and *M. graminicola* lineages. Here, again, one of the gene copies appears to have been lost in *M. graminicola*. The recent duplication of all *H. werneckii* genes presumably results from a whole genome duplication event that occurred after the *M. graminicola*/*H. werneckii* split.

Interestingly, we also observed expansion of the Tok channels in *H. werneckii* (Table S1), since Ion_trans_2 domain (PF07885) in *H. werneckii* was enriched 4× relative to *M. graminicola* and to *S. cerevisiae*. We identified 4 homologues of the Tok1 K^+^ channels (Table 2), each containing two conserved transmembrane helices (PF07885 domain) typical of the ion channel family. Again, sequence identity to the Tok1 protein is low, but the identity is high in both PF07885 domains, with values from 33% to 45.2%, depending on the *H. werneckii* homologue. According to the phylogenetic analysis (Figure 4A) one duplication of Tok genes occured after the separation of *H. werneckii* and *S. cerevisiae* ancestors, but before the separation of *H. werneckii* from *M. graminicola.* In the latter one copy was later lost.

We identified 8 homologues of the Nha1 Na^+^/K^+^, H^+^ antiporter (Table 2), each containing a transmembrane region (PF00999) at the N-terminal, which is conserved through the Na^+^/K^+^, H^+^ exchanger family, and only two of them additionally containing the C-terminal cytoplasmic region (PF08619), with little similarity across the family. Similar to previous transporters, sequence identity to the Nha1 is low, but the aa identity in the conserved PF00999 domain is high, reaching up to 64% in some *H. werneckii* homologues. Several ancient duplications can be seen in the inferred phylogeny of Nha proteins, of which only one copy has been preserved in the genome of *S. cerevisiae* (Figure 4A). This was also confirmed by the comparative analyses of the PFAM domains (Table S1), where PF00999 domain is enriched 5× in *H. werneckii* relative to *S. cerevisiae*, whereas the 2× enrichment of *H. werneckii* relative to *M. graminicola* can be explained by the WGD event.

In contrast with the above transporter families, only 4 homologues of three *S. cerevisiae* Ena Na^+^ P-type ATPases were identified in *H. werneckii* (Table 2). Each homologue contained all 4 conserved domains found in *S. cerevisiae* Ena proteins: the Cation_ATPase_N (PF00690) and Cation_ATPase_C (PF00689) domains, representing the conserved N-terminal and C-terminal region found in H^+^, Na^+^, Ca^2+^, Na^+^/K^+^ and H^+^/K^+^ transporting P-type ATPases; the E1-E2ATPase domain (PF00122), representing the actuator domain and some transmembrane helices found in P-type ATPases; and the Hydrolase_like2 domain (PF13246), a putative hydrolase of sodium-potassium ATPase alpha subunit. The aa sequence identity of the four *H. werneckii* homologues to Ena1 was highest in the PF00122 domain, with values between 44.2% to 52.9%. *S. cerevisiae* Ena1, Ena2 and Ena5 result from recent duplications, while Ena proteins in *H. werneckii* and *M. graminicola* have duplicated much earlier and diverged substantially (Figure 4A). The recent duplication of *H. werneckii* genes is the consequence of the WGD, as in all other cases.

Extensive expansion was also observed for the Pho89 homologues in *H. werneckii*, since the PHO4 domain (PF01384), typical for the phosphate transporter family, was enriched 5× relative to *M. graminicola* and 10× relative to *S. cerevisiae* (Table S1). We identified 6 homologues of the Pho89 Na^+^, P_i_ symporter in *H. werneckii* (Table 2), each homologue containing at least one PHO4 domain (up to two). The aa identity in the N-terminal PF01384 domain was from 44.2 up to 54.7%, depending on the homologue. Pho89 proteins of *H. werneckii* are the result of several duplications that occurred at various times after the separation of its ancestor from the lineage of *S. cerevisiae* (Figure 4A).

Together these observations support the conclusion that for *H. werneckii*, regulation of transport of K^+^ and Na^+^ across the plasma membrane is of utmost importance, because most homologues of the *S. cerevisiae* plasma membrane Na^+^ and K^+^ transporters are enriched in this fungus. This is not unexpected in the case of transporters involved in uptake of K^+^ and efflux of Na^+^(HwTrk, HwNha), which help to maintain a high intracellular K^+^/Na^+^ ratio together with low concentrations of Na^+^ in hypersaline environments [36]. However, enrichments were also observed for transporters responsible for K^+^ efflux and Na^+^ intake (HwTok, HwPho). One reason for this could be the need of *H. werneckii* to quickly adapt to highly dynamic concentrations of NaCl (and other salts) typically encountered by the fungus in its natural environment. When NaCl concentrations are high, import of K^+^ and export of Na^+^ are crucial for survival of the organism. Yet if the concentration of NaCl suddenly drops, the competition between K^+^ and Na^+^ for import into cells ceases to exist, and K^+^ could accumulate to excessive intracellular concentrations [104]. To avoid these consequences, cells must export K^+^ quickly, until K^+^ homeostasis is achieved and the plasma membrane potential restored.

Also of interest is the difference in numbers of *H. werneckii* Nha and Ena homologues. While the first are substantially enriched, this was not observed for the latter. Both proteins export Na^+^ from the cells, but Nha proteins are active at slightly acidic conditions, whereas Ena proteins work in alkaline environments [105]. In laboratory conditions, growth of *H. werneckii* is accompanied by rapid acidification of the medium; however, pH in the solar salterns is typically neutral to slightly alkaline. Although *H. werneckii* could potentially acidify its surroundings in some microenvironments, this cannot fully explain the observed HwNha transporter enrichment. The increase in HwNha numbers may therefore be important for the increase of transcript and protein numbers, as *NHA* gene expression is generally constitutive and very low [105]. Regulation of the transcript numbers of the *ENA* genes on the other hand, is known to occur at the transcriptional level in *S. cerevisiae* [48], [49], [106]. Alkaline pH and increased salinity were shown to be inducers of transcription also in *H. werneckii* [24], which might reduce the need for regulation of expression with gene copy number, as was observed for the *HwNHA*. The HwNha enrichment could also provide the material for evolution of some paralogues towards changed specificity (for Na^+^ or K^+^) or even specialization for other functions. In *S. cerevisiae*, Nha1 namely also has a role in regulation of intracellular pH, cell cycle, cell volume and membrane potential [105], [107]–[109].

We can only speculate on the role of the Na^+^, P_i_ symporter enrichment in *H. werneckii* (HwPho1–3). Pho89 in *S. cerevisiae* utilizes the Na^+^ gradient for import of P_i_ into the cell [110]. Interestingly, PHO89 gene transcription is not induced only by P_i_ limitation, but is strongly induced also by alkaline pH, even when cells are grown in medium with normal phosphate concentrations [111]. Induction by alkaline pH seems to be largely dependent on calcineurin [112]. We propose that in *H. werneckii*, in conditions of high salinity and alkaline pH, where H^+^ gradient cannot efficiently energise the P_i_ import, Na^+^ gradient could represent an alternative energy source for transport.

No homologues of the K^+^, H^+^ symporter Hak1, mediating high affinity K^+^ uptake, or Na^+^ uptake, as is the case in *Yarrowia lipolytica* [113]; or the P-type ATPase Acu, mediating high affinity K^+^ or Na^+^ uptake, were found in *H. werneckii*. Its K^+^ management system therefore differs substantially compared to the closely related *M. graminicola*, which has both Hak1 and Acu homologues [61]. *M. graminicola* also has only one Trk and one Tok channel, compared to 8 and 4 copies of each in *H. werneckii*, respectively. It appears that *M. graminicola* requires active import of potassium, while in *H. werneckii* passive transport is more pronounced. The difference in the transporter inventories of closely related *H. werneckii* and *M. graminicola* possibly reflect the living styles of these fungi, one being an extremely halotolerant fungi and the other a plant pathogen.

#### Intracellular cation transporters

Maintenance of K^+^ homeostasis and sodium detoxification in the cytosol is connected to the cation transport across organellar membranes, which are important for the regulation of organellar pH and volume [43]. In *S. cerevisiae*, endosomal Nhx1 [52] and Kha1 from the Golgi apparatus [53] are typical Na^+^, H^+^ exchangers, similar to the plasma membrane Nha1 [50]. Vacuolar Vnx1 [51] and mitochondrial Mdm38 and Mrs7 [54], [55] have similar Na^+^/K^+^, H^+^ exchanger functions, but different structures.

Homologues of the Nhx1 and Kha1 are duplicated in the *H. werneckii* genome, as a consequence of the WGD event (Table 2 and Figure 4B). All homologues contain the Na_H_Exchanger domain (PF00999) typical for the sodium/hydrogen exchanger family, but lack the C-terminal cytoplasmic region (PF08619) found in Nha1. The aa sequence identity between HwNhx1A, HwNhx1B and Nhx1 is especially high in the conserved PF00999 domain (up to 62.2%). Similar relationships were observed for the Kha1 homologues, where identity in the PF00999 domain is around 47%. We identified two homologues of the human LETM1 transporter in *H. werneckii*, with high sequence identity to the Mrs7 and Mdm38 transporters from *S. cerevisiae* (Table 2 and Figure 4B). Both *H. werneckii* Mrs7 (and Mdm38) homologues contain the LETM1 conserved domain (PF07766), which have 50% aa identity when compared to the Mrs7 domain.

Of the intracellular cation transporters, only the homologues of the vacuolar Vnx1 are enriched in *H. werneckii* relative to the *S. cerevisiae* (4×) (Table S1). We identified 8 homologues of the Vnx1 Na^+^/K^+^, H^+^ antiporter (Table 2), each containing two PF01699 domains, otherwise typical for the sodium/calcium exchanger protein family, but in the case of Vnx1 involved in the Na^+^/K^+^, H^+^ exchange [51]. The enrichment is the result of an ancient duplication before the separation of *H. werneckii* and *S. cerevisiae* ancestors, with one of the lineages, which was lost in *S. cerevisiae*, leading to three copies in *M. graminicola* and six in *H. werneckii* through several additional duplications (Figure 4B). The sequence identity of the HwVnx proteins compared to Vnx1 is low, but the aa sequence identity in the conserved PF01699 domains is up to 70.2% for the first and up to 55% for the second domain, the value also depending on the HwVnx homologue type. Interestingly, the enrichment of the Vnx1 homologues relative to *S. cerevisiae* is observed for both, *H. werneckii* and *M. graminicola*. The abundance of vacuolar Na^+^/K^+^, H^+^ antiporters in *H. werneckii* could contribute to the fungus highly haloadaptable character, probably by accumulating Na^+^ in the vacuoles and thereby helping in detoxification of the cytosolic Na^+^. The role of Vnx transporter enrichment in *M. graminicola* is unkown, but would be interesting to study.

#### Plasma membrane and vacuolar H^+^ ATPase

The activities of many transporters are essential for maintaining the gradient of protons across membranes, generated in large part by the Pma1 P-type ATPase at the plasma membrane [56], [57] and V-type ATPase at the vacuolar membrane [58]. As *H. werneckii* shows itself to have a complex cation transporter system, we analysed the transporters responsible for supplying the needed energy in further detail.

**Figure 5:**
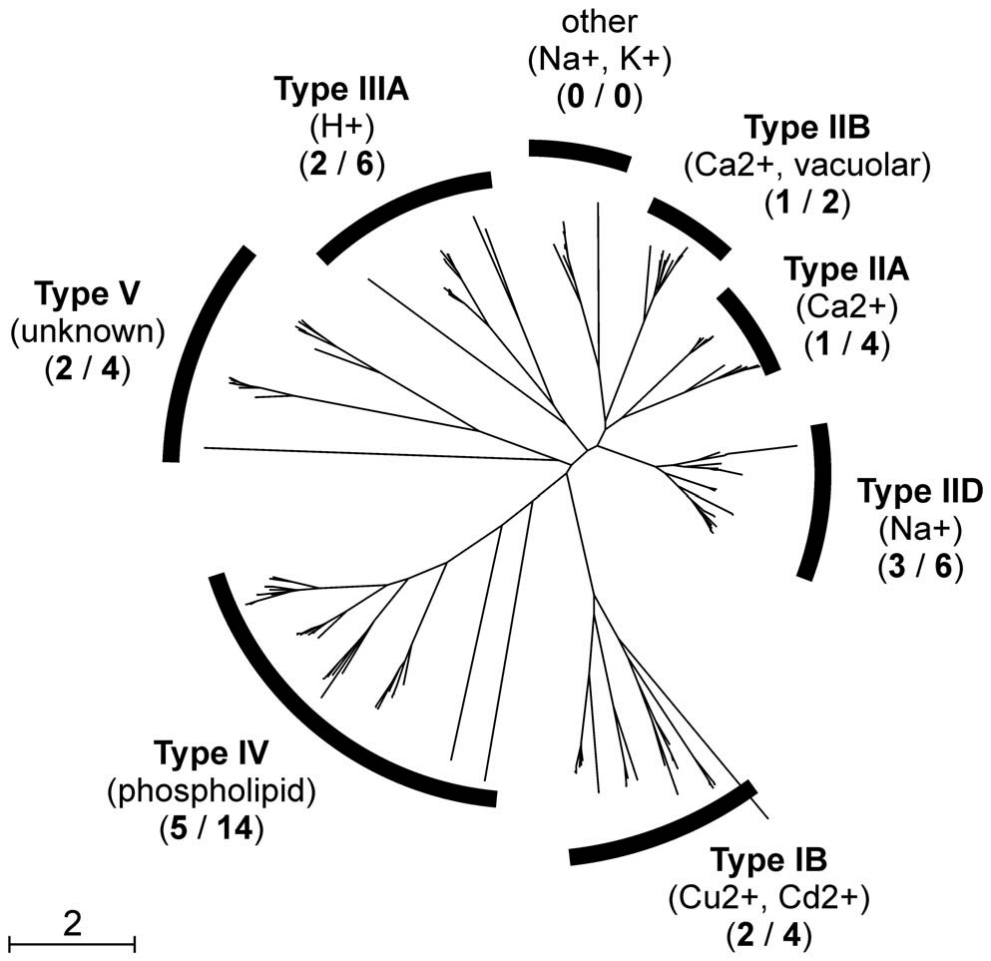
Gene tree of P-type ATPases from *Hortaea werneckii*, *Ajellomyces dermatitidis, Leptosphaeria maculans*, *Mycosphaerella graminicola*, *Paracoccidioides brasiliensis,* and *Saccharomyces cerevisiae.* The specificity of each group of proteins is described in brackets immediately below the name of the group. Below this, numbers of proteins belonging to a given group in the proteomes of *S. cerevisiae* and *H. werneckii* (Sc/Hw) are shown, respectively.

We identified four homologues of Pma1 in *H. werneckii*; HwPma1A (46% identity to Pma1), HwPma1B (46.1%), HwPma2A (45.6%) and HwPma2B (45.9%) (Table 3). Each homologue containes 3 conserved domains also found in *S. cerevisiae* Pma1 and Pma2 proteins: the Cation_ATPase_N domain (PF00690), the E1-E2ATPase domain (PF00122) and the Hydrolase_like2 domain (PF13246). Similar domains are also typical for other members of the P-type ATPase family, such as the previously mentioned Ena proteins. Generally, P-type ATPases have a common mechanism of action – hydrolysis of ATP to energise the transport of different ions and other substrates through the membrane; therefore they have a similar structure [114]. Based on the reconstruction of their phylogeny, they have been classified into five families and further into two or more subfamilies [115]. The number of P-type ATPases in different species is highly variable [116]. By searching the predicted *H. werneckii* proteome with blastp (e-value cut-off 10^−6^) using P-type transporters from *S. cerevisiae* as queries, we identified 40 members of the P-type family in *H. werneckii* (Figure 5). The number of proteins was twice as large in *H. werneckii* as in *S. cerevisiae* in the case of groups IB (transport of Cu^2+^, Cd^2+^ and other metals), IIB (Ca^2+^ transport), IID (Na^+^ transport), and V (pumps with unknown function). The following groups were even more enriched in *H. werneckii*, group IIA (Ca^2+^ transport) having 4 members, group IIIA (proton transport) 6 members and group IV (phospholipid transport) 14 members (Figure 5).

**Figure 6:**
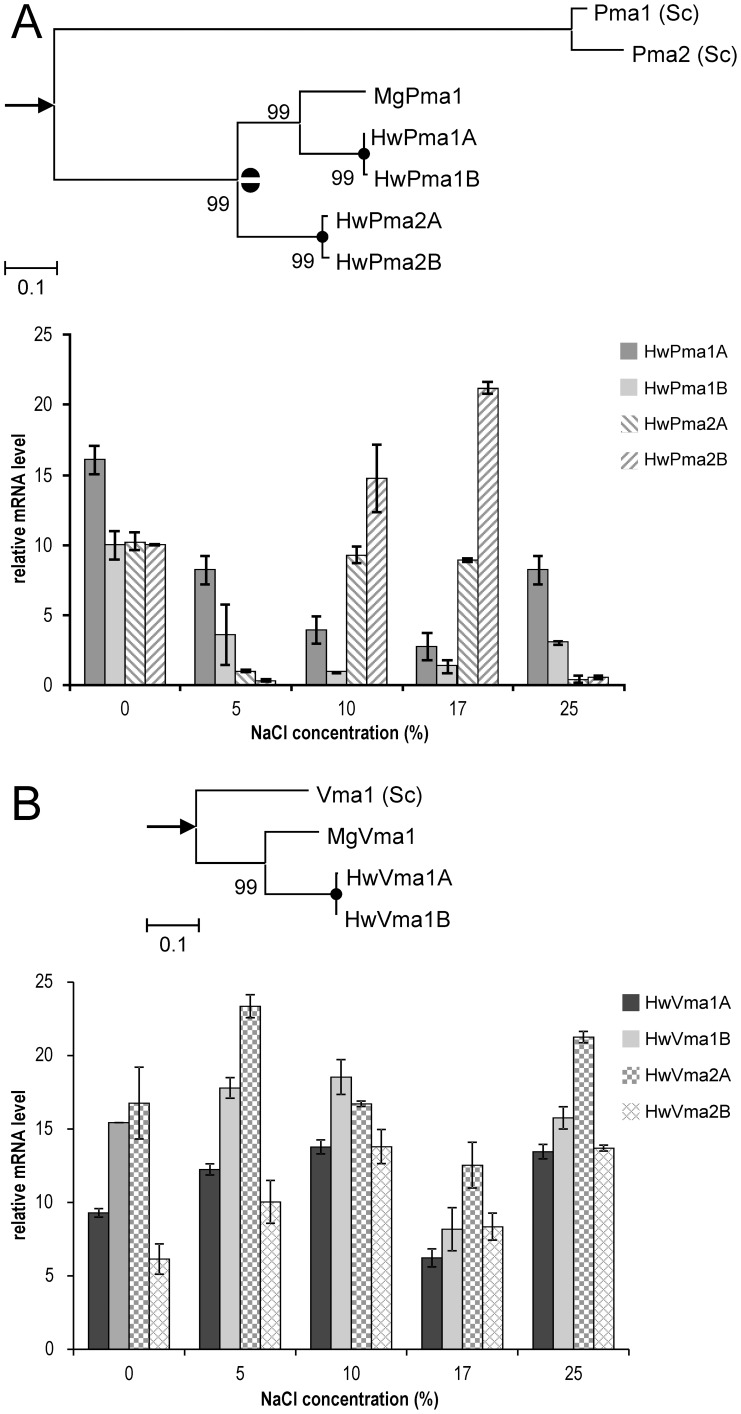
Proton ATPases. **A.** Plasma membrane ATPases (Pma). Gene phylogeny (above) of homologues from *Hortaea werneckii* (HwPma), *Mycosphaerella graminicola* (MgPma1: XP_003852209.1) and *Saccharomyces cerevisiae* (Pma1: YGL008C, Pma2: YPL036W), rooted by a homologue from *Cryptococcus neoformans* (XP_568571.1). Two half-circles mark a duplication after the separation of *S. cerevisiae* and *H. werneckii* ancestors, but before the separation of *H. werneckii* and *M. graminicola*, black circles on the bifurcation mark recent duplications presumably resulting from a whole genome duplication. Transcription profiles (below) of plasma membrane H^+^-ATPases of *H. werneckii* homologues at different concentrations of NaCl (w/v). Quantitative reverse transcription PCR (qRT-PCR) was performed with RNA isolated from cells grown in YNB medium, supplemented with 0, 5, 10, 17, and 25% NaCl (w/v). Quantification cycle (Cq) values for our genes of interest were normalised to the quantification cycle of 28S rRNA fragment (reference gene). The difference in Cq values (relative mRNA level values) between the target gene and the reference gene was calculated, and these values of the different samples were compared directly. Data are means of relative mRNA level values obtained by two qRT-PCR experiments performed with biological triplicates. **B.** The subunit A of the vacuolar ATPases (Vma1). Gene phylogeny (above) of homologues from *Hortaea werneckii* (HwVma), *Mycosphaerella graminicola* (MgVma1: XP_003850333.1) and *Saccharomyces cerevisiae* (Vma1: YDL185W), rooted by a homologue from *Cryptococcus neoformans* (XP_570895.1). Black circles on the bifurcation marks a recent duplication presumably resulting from a whole genome duplication. Transcription profiles (below) of vacuolar H^+^-ATPases of *H. werneckii* homologues at different concentrations of NaCl (w/v). Quantitative reverse transcription PCR (qRT-PCR) experiment and the analysis of the data was performed as described above.

The analysis of *H. werneckii* Pma-family proton pumps showed that apart from the gene duplications presumably originating from a WGD, one additional duplication occurred in the evolution of Pma pumps, resulting in two lineages, of which only one survived in the genome of *M. graminicola* (Figure 6A). To investigate the relevance of the four *H. werneckii* Pma homologues for the cation homeostasis, we checked the level of transcription of each gene during growth at different NaCl concentrations (Figure 6A). *S. cerevisiae* has two copies of the gene, but *PMA2* is expressed at such low levels relative to *PMA1*, that it is considered nonessential with only minor impact on the homeostasis [117]. In *H. werneckii* the transcription profiles of the *PMA1* and *PMA2* homologues show responsiveness to different salinity conditions, with the lowest transcription of *PMA1* homologues at a salinity of 17% NaCl (w/v), where the growth starts to slow down [25]. This result corresponds to the findings of Vaupotič and Plemenitaš [33] where the lowest transcription of *H. werneckii PMA* homologue (sequence corresponding to our gene *HwPMA1B*) was detected when the *H. werneckii* cells were grown at 3 M (17.5%) NaCl in comparison to the transcription at 4 M (26%) NaCl. The *H. werneckii PMA2* homologues had the lowest transcription at optimal salinity of 5% NaCl and at 25% NaCl. The low *PMA2* transcription at 25% NaCl, where the stress becomes more severe, can then be partially replaced by the transcription of *PMA1* homologues. Comparisons of the transcription profiles of *PMA* genes in *H. werneckii* with those described in *S. cerevisiae* showed that in *S. cerevisiae PMA1* was not induced by salt stress [118]. In *H. werneckii* both *PMA1* and *PMA2* manifest salt-regulated transcription. These gene transcription measurements are suggestive of function in halotolerance but do not consider the impact of post-translation modification on activity.

In addition to its critical role(s) in acidification of the vacuolar lumen, the yeast vacuolar ATPase is also important for the proper functioning of other organelles [43]. It has a complex structure, consisting of the peripherally associated catalytic V_1_ subcomplex, comprised of proteins coded by 8 different genes (*VMA1*, *VMA2*, *VMA4*, *VMA5*, *VMA7*, *VMA8*, *VMA10*, *VMA13*), and a proton translocating membrane V_0_ subcomplex comprised of proteins coded by 6 different genes (*VPH1*, *STV1*, *VMA3*, *VMA6*, *VMA11*, *VMA16*) [58]. *H. werneckii* codes for homologues of all subunits of the *S. cerevisiae* V-ATPase complex, which are always duplicated as a consequence of the WGD event (Table 3). The *H. werneckii* V_1_ subunits (Table 3) in general share a lot of similarity with the *S. cerevisiae* subunits (Figure 6B), which is not surprising, as their structure and function have been highly conserved through evolution [58]. One of the most conserved subunits are the subunit A (Vma1) and B (Vma2), which form the catalytic and nucleotide-binding subunits [58]. The overall aa sequence identity between *H. werneckii* subunit A homologues and Vma1 is 48.6%, reaching 73.5% if compared to the sequence of the final product of Vma1, which is lacking the intein sequence. The intein sequence, typical for *S. cerevisiae* [119], is namely missing in the *H. werneckii* Vma1 homologue, and also in the related *M. graminicola*. Interestingly, both *H. werneckii* (*HwVPH1A*, *HwVPH1B*) and *S. cerevisiae* (*VPH1*, *STV1*) code for two homologues of the V_0_ subunit a (Table 3). Vph1 in yeast is a part of the vacuolar membrane V-ATPase; whereas its homologue Stv1 is part of the V-ATPase found on the Golgi or endosome membranes [58]. Vph1 and Stv1 have 49.4% identity in the aa sequence, whereas HwVph1A and HwVph1B have 99.5% aa identity. It is therefore unlikely that HwVph1A and HwVph1B proteins would localise to different cellular locations, as is observed for the *S. cerevisiae* homologues. It remains to be resolved how *H. werneckii* specifically localises the ATPase complexes to the vacuoles or to the Golgi or endosome membranes. Although the *HwPMA*s transcription is salt-regulated, no significant trends were seen for the transcription of *VMA* homologues under different salinities (Fig. 5B). In contrast in *S. cerevisiae* salt stress induces the transcription of vacuolar ATPase subunits [118].

#### Promising *Hortaea werneckii* genes for improvement of crop salt tolerance

Many cation transporters have been conserved throughout evolution, therefore similar transporters found in the *S. cerevisiae*, *H. werneckii* and other fungi are also found in plants. For example, Quintero et al [38] showed that expression of the *Arabidopsis thaliana* AtNHX1 protein functionally substituted the endosomal Na^+^/H^+^ antiporter function lacking in the *nhx1 S. cerevisiae* mutant. Another study similarly showed that the *nhx1 S. cerevisiae* mutant could be used for selection of AtNHX1 proteins, which were improved for salt tolerance by random mutagenesis [40]. Quintero et al [39] have also successfully reconstituted the *A. thaliana* SOS signalling pathway in the yeast. This pathway is essential for Na^+^ homeostasis and is composed of the ion transporter SOS1, protein kinase SOS2 and the Ca^2+^ sensor SOS3.

Recently, it was shown that cell type specific expression of the Na^+^ transporter AtHKT1 [42] or overexpression of the plasma membrane Na^+^/H^+^ antiporter SOS1 [41] in *Arabidopsis thaliana* improved the plant salt tolerance. The above mentioned studies have only focused on plants as the source of transgenes for improvement of crop resistance to high salinity environments. As shown by this study, enrichment in cation transporters important for maintaining K^+^ homeostasis, low Na^+^ content and pH homeostasis, are crucial for *H. werneckii* survival in environments with rapid changes of NaCl concentrations. Therefore these genes could be interesting novel candidates for improving the halotolerance of plants. Testing new approaches for this purpose is of great importance, since past considerable efforts with various targets (genes encoding enzymes involved in compatible solute synthesis, antioxidants heat-shock and late embryogenesis abundant proteins, and transcription factors for gene regulation), did not yet produce crops with satisfactory improvements in salt tolerance under field conditions [4], [120], [121].

Sodium is toxic to plant cells, because it competes with K^+^ for binding sites involved in activation of at least 50 cytoplasmic enzymes [122]. Central to salt tolerance is therefore the reduction of Na^+^ toxicity, which can be achieved by restricted Na^+^ uptake, active Na^+^ exclusion or compartmentalization of excessive Na^+^ in the vacuole. Although some improvements of plants were done in this direction [41], [42], *H. werneckii* Nha1 and Vnx1 homologues are very interesting novel targets for plant transgenes. In *H. werneckii* low amounts of cytosolic Na^+^ were observed over the whole range of salinities [25], indicating very efficient exclusion mechanisms, possibly due to action of export from the cell by HwNha proteins, and effective accumulation into vacuoles by HwVnx proteins. Due to both large copy numbers and great diversification of HwNha and HwVnx proteins, it would be interesting to test them in the *nha1* and *vnx1 S. cerevisiae* mutants to see if any of the homologues have evolved to confer greater specificity towards Na^+^ exclusion and consequently greater halotolerance. Such modification has already been reported for plant transporters. For example, it was suggested that the difference in salt sensitivity between bread wheat and durum wheat is in enhanced K^+^/Na^+^ discrimination [123]. A similar experiment was done when testing AtNHX1 random mutagenesis mutants in *nhx1* strain by Hernández et al [40].

Although exclusion of Na^+^ is essential, the key determinant of salt tolerance is the ability to support a high intracellular K^+^/Na^+^ ratio and not the absolute quantity of Na^+^ in the cell. Environmental Na^+^ competes with K^+^ for uptake sites of the transporters at the plasma membrane, and causes membrane depolarization [36], [124]. The functionality of the polarization channels is disturbed and consequently, the passive K^+^ uptake is diminished, whereas K^+^ efflux through the channels is increased [36]. Therefore restoring the K^+^ homeostasis is crucial for the halotolerance of the plant, as was already suggested by Horie et al. [125], since constitutive expression of rice Na^+^ insensitive K^+^ transporter, OsHAK5, in cultured tobacco BY2 cells enhanced the accumulation of K^+^ and conferred increased salt tolerance to the cells. It would be interesting to test how *H. werneckii* Trk1 uptake and Tok1 efflux channels function in plant cells exposed to high salinity. The absence of transporters for active import of K^+^ (like Hak1 and Acu) suggests that this fungus achieves homeostasis by specialization of some of its numerous homologues of HwTrk and HwTok. An efficient K^+^ management strategy would also be very beneficial in plants. The salt-tolerant *Thellungiella halophile,* for example, is capable of increasing K^+^ content under saline conditions, in contrast to the decline of K^+^ observed in related *A. thaliana* [126].

The cellular response to hypersaline stress is energetically very demanding, reducing the ATP pools of the cells. Importantly the H^+^ gradient generated by the plasma membrane and vacuolar H^+^-ATPase is crucial to energise secondary cation transport systems [43]. Understanding the regulation and functioning of the *H. werneckii* Pma and vacuolar H^+^-ATPase homologues will shed light on this crucial adaptation processes. However, because of their diverse roles, these genes are not very suitable candidates for transgenes. H^+^ ATPases in plants are namely involved in many physiological functions, like mineral nutrition in the root, metabolite translocation, regulation of cytoplasmic pH, and cell turgor-related functions, such as organ movement and cellular growth [127].

### Conclusions

The *Hortaea werneckii* genome sequence presented here shows many features consistent with adaptation to its unique lifestyle and saline tolerance. Two features in particular stood out in analysis: its large genetic redundancy, presumably resulting from an evolutionarily recent whole genome duplication and the expansion of families of genes encoding metal cation transporters.

Ploidy levels of fungal species can be very fluid [84], [85] and therefore the fact that *H. werneckii* has undergone a recent WGD is not necessarily unique. The fact that duplication has not yet been followed by selective gene loss is however, of considerable interest from both a biological and a biotechnological perspective. Interestingly, experiments on *S. cerevisiae* revealed a tendency toward increased genome size as a response to stressful concentrations of salt [84], [89]. Such redundancy may be an excellent reservoir of cryptic genetic variability, which is of importance in stressful environments that require good adaptability [80].

In hypersaline environments regulation of the transport of K^+^ and Na^+^ across the plasma membrane is of utmost importance. In *H. werneckii* this is seen from the fact that most homologues of the *S. cerevisiae* plasma membrane Na^+^ and K^+^ transporters (e.g. Trk1, Trk2, Tok1, Nha1 and Pho89) are enriched in this fungus. Especially surprising is the enrichment of the Na^+^, P_i_ symporter (HwPho1–3) in *H. werneckii*. Since in *S. cerevisiae PHO89* gene transcription is induced by alkaline pH regardless of environmental phosphate concentrations [111] we propose that in *H. werneckii*, in conditions of high salinity and alkaline pH, Na^+^ gradient could energise the P_i_ import instead of the H^+^ gradient. No homologues of the K^+^, H^+^ symporter Hak1 and the P-type ATPase Acu, important for K^+^ homeostasis in a closely related *M. graminicola*, were found in *H. werneckii*. The enrichment in plasma membrane cation transporters is accompanied by transcript enrichment of the plasma P-type H^+^ ATPases in *H. werneckii*, as transcription of all four *HwPMA*s is salt dependent. In *S. cerevisiae*, only *PMA1* is transcribed in sufficient quantities to substantially contribute to the generation of the proton gradient [117]. The difference in the transporter inventories of the mesophylic *S. cerevisiae*, a plant pathogen *M. graminicola*, and its closely related extremely halotolerant *H. werneckii* does not only reflect their phylogenetic relations, but in an even greater extent also their diverse life styles. Therefore, the great diversity of *H. werneckii* cation transporters and their possibly novel adaptations to high concentrations of salt may harbour a great biotechnological potential for improving the halotolerance of salt-sensitive species. In *H. werneckii*, low amounts of cytosolic Na^+^ were observed over the whole range of salinities [25], indicating very efficient plasma membrane Na^+^ exclusion and vacuole Na^+^ import mechanisms, possibly through action of HwNha and HwVnx proteins. This makes *H. werneckii* Nha1 and Vnx1 homologues especially interesting targets for plant transgenes.

The genetic redundancy and enrichment of cation transporters seem to be at the core of the extremely halotolerant phenotype of *H. werneckii*. Thorough understanding of eukaryotic halotolerance is important for alleviating problems such as those caused by soil salinization in agriculture or osmotic stress in the production of bioethanol. *H. werneckii* is an appropriate model organism for studying an excellent ability for adaptation to almost the whole range of salinities. While working with it in the past was challenging, the availability of the genomic sequence should significantly ease further studies of this exceptional species.

## Supporting Information

Figure S1 **Distribution of predicted proteins from** ***Hortaea werneckii*** **across different gene ontology categories.**
(EPS)

Table S1 **Number of proteins of** ***Hortaea werneckii*** **with a specific PFAM domains.**
(XLS)

Table S2 **Primers for quantitative reverse transcription PCR.**
(DOC)
