# Supplementary material for: Whole Genome Duplication and Enrichment of Metal Cation Transporters Revealed by De Novo Genome Sequencing of Extremely Halotolerant Black Yeast Hortaea werneckii
Source: PLoS One. 2013 Aug 15;8(8):e71328. doi: 10.1371/journal.pone.0071328 (PMC3744574; doi:10.1371/journal.pone.0071328)
Supplement: Table S2 — Primers for quantitative reverse transcription PCR. (DOC) [file pone.0071328.s003.doc]

Supporting Table S2. Primers for quantitative reverse transcription PCR.

| **Gene Name** | **Accession No.** | **T annealing** | **Primer** | **Sequences (5′ to 3′)** |
| --- | --- | --- | --- | --- |
| *HwPMA1A* | KC961362 | 60°C | HwPma1A-F | GTTGACATGCCACTTCATC |
|  |  |  | HwPma1A-R | TTCATCGTTGCCACCCTCA |
| *HwPMA1B* | KC961363 | 60°C | HwPma1B-F | TTGTTGACACGCCACTCTGTT |
|  |  |  | HwPma1B-R | TTCATCGTTGCCGCCCTCG |
| *HwPMA2A* | KC961364 | 55°C | HwPma2A-F | ACCTTGCAATCGGGCCATCG |
|  |  |  | HwPma2A-R | TGACTGTTCGCCGCCAACC |
| *HwPMA2B* | KC961365 | 50°C | HwPma2B-F | ACCTCGTCATTGGGTCACCA |
|  |  |  | HwPma2B-R | GAATCTTGGCCACCAATG |
| *VMA1A* | KC961366 | 60°C | HwVma1A-F | ACGGAGAGGAGAAGGTGGGCA |
|  |  |  | HwVma1A-R | CTTTCATCATCGCCGCGCAC |
| *VMA1B* | KC961367 | 60°C | HwVma1B-F | ATGGCGAGGAGAAGGTGGGTA |
|  |  |  | HwVma1B-R | GTTTCATCGCCGCGCACTG |
| *VMA2A* | KC961368 | 55°C | HwVma2A-F | AAGGGAACGGAGGACA |
|  |  |  | HwVma2A-R | AAGGAGAACTGGAAAGAG |
| *VMA2B* | KC961369 | 55°C | HwVma2B-F | AAGGGAACGGAGGATA |
|  |  |  | HwVma2B-R | CAAAGAGAACATCAAAAGAG |
| *Hw28SRR* | ---------------- | 60°C | Hw28S-F | TCGACGAGTCGAGTTGTTTG |
|  |  |  | Hw28S-R | CTTGTGCGCTATCGGTCTCTG |
